# Acute Rheumatism; Pericarditis; Sudden and Rapid Effusion into Pericardium; Paracentesis Pericardii; Recovery

**Published:** 1874-12-01

**Authors:** 


### ACUTE RHEUMATISM; PERICARDITIS; SUDDEN AND RAPID EFFUSION INTO PERICARDIUM; PARACENTESIS PERICARDII; RECOVERY.

*From London Lancet, November*, 1874.

W. L———, aged twenty-five, married; a plasterer. Has had four attacks of "rheumatic fever" during the last three years, but does not know that his heart was affected in any way. Has not complained of shortness of breath, and has had no difficulty in getting about. Has been ailing for the last nine months, but did not lie up till three weeks ago, when pain in the hips came on, and he has been obliged to remain in bed since.

Admitted March 13th, 1874. Complains of feeling sore all over. He is unable to move without pain, but no articular swelling is visible. Perspires freely. Has a depressed expression, and lies on his back. Temperature 102-6°; pulse 108; respiration 32. Complains of pain in the cardiac region on drawing a deep breath. A friction sound is audible over the base of the heart, and a prolonged blowing sound at the apex. There is no increase of cardiac dullness. The urine has a specific gravity of 1025; it is slightly acid, but free from albumen. Ordered six leeches to be applied to the cardiac region, and to take an alkaline mixture every four hours.

14th.—Friction sound at the base less distinct. Temperature 101-2°F.; pulse 92; respiration 28. There is slight effusion into both knee-joints, and tenderness about the ankles. The precordial pain relieved.

23d.—The friction sound still audible. Ordered some blistering fluid to be applied over the base of the heart.

24th.—A loud double friction sound audible over the whole cardiac area, but no increase of cardiac dullness.

25th.—Was taken suddenly in the night with acute precordial pain and dyspnœa. At midday the pain was unrelieved, and dyspnœa considerable. Pulse small and weak, 130; respiration 44; temperature 100° F. Cardiac dullness extends to the second interspace; no cardiac impulse to be felt; heart's sounds scarcely audible; no friction sound. Lips, fingers, and toes looking blue; no impairment of consciousness, but dyspnœa very urgent. Paracentesis pericardii was considered necessary, as nothing short of the removal of the fluid from the pericardium seemed likely to restore the rapidly weakening power of the heart. Accordingly Mr. Charles Steele was called in consultation, and immediately proceeded to perform the operation. One of the larger sized tubes of Dieulafoy's aspirator was plunged through the skin and chest-wall at a spot between the fourth and fifth ribs and half way between the middle line and the nipples on the left side. Several ounces of perfectly clear serous fluid were then drawn off by suction, but the fluid gradually became more and more colored till it appeared to be mere blood. After ten ounces of fluid had been withdrawn, the tube was removed, and the aperture closed with strapping. The area of cardiac dullness had considerably diminished and the dyspnœa was much relieved for a few minutes, but the dullness increased again in about ten minutes, though not to the same extent as before the operation, and he still had considerable difficulty in breathing, but the pulse was stronger than before. It was presumed that some hæmorrhage into the pericardium had taken place, as the last few ounces of fluid looked like undiluted blood, and the whole quantity of fluid became a coherent mass of coagulum after standing. In the evening he breathed more easily. The pulse was 124, fuller than before the operation; respiration 44; temperature 103° F.

26th.—Pulse 124; respiration 36; temperature 101°, morning.

28th.—Pulse 116; respiration 32; temperature 100. The left wrist was painful and swollen; he perspired very copiously; pericardial dullness less; respiration easy.

On April 2d he was free from pain and slept well. On the 7th he was not so well. Pulse 112; temperature 99-6°. Did not complain of pain; could draw a deep breath without difficulty; cardiac dullness normal; a slight systolic friction sound audible at the base of the heart.

On May 19th he was discharged, the heart's sounds being normal, the area of cardiac dullness not enlarged. He was still weak, and the pulse was rather small, soft and quick.

On July 6th he was doing his regular work without much difficulty.

---

IODIDE OF POTASSIUM IN ACUTE AND CHRONIC BRONCHITIS AND ASTHMA (*The British Medical Journal*, September 5, 1874).—Mr. W. H. Spurgiss has used iodide of potassium in over one hundred cases of the above diseases, and with invariable success, now rarely giving anything else.

GALVANO-PUNCTURE AT THE WESTMINSTER HOSPITAL.—On Tuesday last (July 14th) the operation of galvano-puncture was performed on a man aged thirty-eight, under the care of Dr. Anstie, for an abdominal aneurism supposed to be connected with the cæliac axis. The patient first noticed a pulsating swelling in the left hypochondriac region ten weeks ago. A fortnight afterward he applied at the hospital and was admitted, and was kept at rest in bed. The aneurism was at first treated according to the plan recommended by Langenbeck, by the subcutaneous injection of ergotin. Bonjean's ergotin was injected seven times, at intervals of about two days. Altogether, thirty grains were used, but without any definite effects except on the first occasion. Galvano-puncture was therefore determined on. Accordingly, on Tuesday last, two needles, insulated with vulcanite up to three-quarters of an inch of the points, were introduced into the aneurism at the most prominent part, and were then connected with the positive pole of the battery, which consisted at first of twelve Bunsen's cells, subsequently reduced to eight. The current was kept up for thirty minutes, during which time the patient suffered acute pain. Both needles were then cut short and left in the aneurism for twenty-three hours, when they were removed on account of the redness and discoloration of the skin. The day after the operation the aneurism was firmer, and the pulsation appreciably diminished, the patient being quiet and composed. A subcutaneous injection of a third of a grain of morphia was given on the night of the operation, and another injection on the next morning. The patient continues in a very satisfactory state, and when the case is more advanced we shall give in detail the ultimate results. —*London Lancet.*

NEW RESEARCHES ON DIABETES.—We learn that Dr. Pavy has obtained some experimental results which are likely to throw a new light on the subject of diabetes. He has found that the injection of defibrinated arterial blood into the portal system occasions a saccharine state of the urine. In one experiment, the urine after the operation contained fifteen grains of sugar to the fluid ounce, and in others the quantity has amounted to nearly the same. In the counterpart experiment of injecting defibrinated venous blood into the portal system, the urine showed no signs of the presence of sugar. It thus appears that oxygenated blood passing to the liver causes an escape of sugar from the organ, and thence an accumulation in the system and discharge with the urine. It also appears that through the medium of the respiration of oxygen he has succeeded in inducing a sufficiently oxgenated state of the blood to similarly give rise to the production of saccharine urine. He has further found that through the agency of the inhalation of puff-ball smoke an immediate and strongly diabetic state may be induced, and that the effect is accompanied with such a modification of the circulation that the blood flows through the vessels, as is the case after section of the sympathetic, without becoming de-arterialized. His experiments, he considers, suggest that in diabetes of the human subject, the blood, in consequence of vaso-muscular paralysis, is allowed to reach the portal vein in an imperfectly de-arterialized condition, and thus determines the escape of sugar from the liver. We understand his results are to be brought forward at the Royal Society as soon as its meetings commence.—*Ibid.*

LOCAL ACTION OF IPECACUANHA (*The Practitioner*, September, 1874). —Dr. De Mussey records a case of purulent ophthalmia in a new-born infant, in which, after all other remedies had failed, he succeeded in producing a cure by the use of the following decoction:

℞ Ipecac root, ʒ ss

Water, ℥ v.

Boil for ten minutes, and, when cool, strain off.

